# Chemotherapeutic Risk lncRNA-PVT1 SNP Sensitizes Metastatic Colorectal Cancer to FOLFOX Regimen

**DOI:** 10.3389/fonc.2022.808889

**Published:** 2022-03-31

**Authors:** Shenshen Wu, Xi Yang, Weiyan Tang, Giuseppe Familiari, Michela Relucenti, Michael Aschner, Xiaobo Li, Rui Chen

**Affiliations:** ^1^ Advanced Innovation Center for Human Brain Protection, Capital Medical University, Beijing, China; ^2^ Beijing Key Laboratory of Environmental Toxicology, Capital Medical University, Beijing, China; ^3^ Department of Occupational Health and Environmental Health, School of Public Health, Capital Medical University, Beijing, China; ^4^ Key Laboratory of Environmental Medicine Engineering, Ministry of Education, School of Public Health, Southeast University, Nanjing, China; ^5^ Medical Oncology, Jiangsu Cancer Hospital, Nanjing, China; ^6^ Department of Anatomical, Histological, Medical and Legal Locomotive Apparatus, Section of Human Anatomy Via Alfonso Borelli, Sapienza University of Rome, Roma, Italy; ^7^ Department of Anatomical, Histological, Forensic Medicine and Orthopedic Science, Sapienza University of Rome, Roma, Italy; ^8^ Department of Molecular Pharmacology, Albert Einstein College of Medicine, Bronx, NY, United States; ^9^ Department of Toxicology and Sanitary Chemistry, School of Public Health, Capital Medical University, Beijing, China

**Keywords:** colorectal cancer, PVT1, polymorphism, chemotherapy, survival

## Abstract

Recent studies have identified that long noncoding RNA (lncRNA) might affect the responses to anticancer drug treatment, including colorectal cancer (CRC). However, the association between single-nucleotide polymorphisms (SNPs) in PVT1 and the chemotherapy response in metastatic colorectal cancer has yet to be clarified. In this study, the PVT1 rs2278176 CT/TT genotypes were found to be associated with an increased overall survival (OS) and progression-free survival (PFS) compared with the CC genotype. Furthermore, patients harboring the rs2278176 CT/TT genotypes had a greater chance of achieving clinical benefit from 5-Fluorouracil/leucovorin combined with oxaliplatin (FOLFOX). *In vivo* nude mice experiments demonstrated that the CRISPR/Cas9 mediated rs2278176 C to T mutation significantly inhibited the tumorigenesis of colorectal cancer cells treated with 5-Fu, but not control DMSO treated cells. Furthermore, the apoptotic rate was significantly enhanced by treatment with 5-Fu in the CRC cells carrying with the CT/TT genotypes. Functional studies demonstrated that the PVT1 rs2278176 C to T mutation altered the binding site for hsa-miR-297, and that hsa-miR-297 downregulated Glutathione S-Transferase Alpha 2(GSTA2), a member of phase II detoxification enzyme, in an Argonaute 2(Ago2)-dependent manner. Moreover, GSTA2 levels were downregulated in the cancer tissues of patients carrying rs2278176 CT/TT genotypes. High GSTA2 expression predicted poor clinical outcome in metastatic colorectal cancer treated with FOLFOX. In conclusion, this study provided that PVT1 with rs2278176 T allele altered the binding affinity with hsa-miR-297, leading to decreased GSTA2 expression and sensitized CRC cells to FOLFOX chemotherapy, suggesting rs2278176 CT/TT genotypes might serve as a predictive biomarker to improve prognosis in patients with metastatic CRC treated with FOLFOX.

## Introduction

Patients with colorectal cancer (CRC) having unresectable distant metastases should be offered palliative chemotherapy (1). 5-Fluorouracil/leucovorin combined with oxaliplatin (FOLFOX) represents one of the most effective chemotherapy regimens for metastatic CRC, with a response rate of more than 40% in the first-line treatment (2). Although FOLFOX has been well recognized as the standard chemotherapy for metastatic CRC, the response to chemotherapy differs among patients with CRC (3–5).

Long noncoding RNAs (lncRNAs) are nonprotein-coding transcripts longer than 200 nucleotides. Emerging evidence has demonstrated that the aberrant expression and function of lncRNAs is often inherent to numerous human diseases, including cancer (6–9). The human PVT1 gene maps to chromosome 8q24 and encodes a lncRNA. PVT1 is documented to be involved in a variety of cancers development and plays a major role in regulating the efficacy of cancer chemotherapy (10–17). PVT1 promotes gemcitabine resistance of pancreatic cancer *via* activating Wnt/β-catenin and autophagy pathway through modulating the miR-619-5p/Pygo2 and miR-619-5p/ATG14 axes (14). Zhang et al. reported that the overexpression of PVT1 might promote multidrug resistance in gastric cancer by upregulating the expression of multidrug resistance–related genes (18). However, traditionally studies mainly focused on the effects of PVT1 on regulating the efficacy of cancer chemotherapy, few research explored the underlined individual difference. Further, it has been shown that single-nucleotide polymorphisms (SNPs), one of the most common genetic variations, might affect the responses to anticancer drug treatment, including CRC (19–21). The possibility that SNPs in PVT1 contribute to the chemotherapy response in metastatic CRC was not clear.

Therefore, this epidemiologic study was conducted to evaluate the effects of the potential functional genetic variants in PVT1 on the chemotherapy response in metastatic CRC and further explore the biological effect of a select number of SNPs.

## Materials and Methods

### Study Participants

A total of 521 patients with metastatic CRC was enrolled in the study, including 170 patients in the Xuzhou cohort and 351 patients in Nanjing cohort from January 2007 to October 2011. The demographic information of patients with CRC between the two cohorts was comparable and shown in **Table 1** and **Table S1**.

**Table 1:** Frequency distribution of the selected variables in colorectal cancer patients.

Variables	Xuzhou Cohort (N=170)		Nanjing Cohort(N=351)		P
	patients	%	patients	%	
Age					0.8788
≤54	85	50.0	173	49.3	
>54	85	50.0	178	50.7	
Gender					0.6019
Male	104	61.2	223	63.5	
Female	66	38.8	128	36.5	
Location					0.1824
colon	112	65.9	210	59.8	
rectum	58	34.1	141	40.2	
Grade					0.0189
low	100	58.8	168	47.9	
Intermediate/High	70	41.2	183	52.1	
Depth of invasion					0.1487
T1	1	0.6	15	4.3	
T2	17	10.0	35	10.0	
T3	27	15.9	50	14.3	
T4	125	73.5	251	71.5	
Lymph node metastasis					0.8101
N0	31	18.2	61	17.4	
N1	139	81.8	290	82.6	
Tumor Response					0.4017
CR+PR	102	60.0	197	56.1	
SD+PD	68	40.0	154	43.9	

CR, complete response; PR, partial response; SD, stable disease; PD, progressive disease.

In addition, a total of 20 pairs of CRC tissues and the corresponding adjacent noncancerous tissues, which were used to evaluate GSTA2 levels in patients with different rs2278176 genotypes, were obtained from patients undergoing surgery in the Jiangsu Tumor Hospital between 2014 and 2015.

### Data Collection and Follow-Up

All patients with CRC were treated with FOLFOX-based chemotherapy. Tumor response was evaluated after four cycles of treatment and every four cycles until the time of death or the first observation of disease progression according to the Response Evaluation Criteria in Solid Tumors (22). All patients were followed up by personal or family contacts until the time of death or last follow-up (June 2016).

### SNP Selection

SNPs in PVT1 gene were selected from the LncRNASNP database (23). The two SNPs (rs11604 and rs2278176) to be evaluated were selected based on the following three filtering criteria (**Figures S1A**–**C**):

1. Minor allele frequency was >0.1 in global and the Han Chinese in Beijing, China (CHB)populations from the 1000 Genomes Project.
2. SNPs in PVT1 gene might destroy or create miRNA-binding sites on lncRNAs.
3. The secondary structure changed with RNAfold (**Figures S1B, C**).

(http://rna.tbi.univie.ac.at/cgi-bin/RNAWebSuite/RNAfold.cgi).

### Genotyping

The genotypes of selected polymorphisms were determined by TaqMan allelic discrimination method using the Quant Studio 6 Flex system (Applied Biosystems, CA, USA). Approximately 10% of the random samples were selected to repeat the genotyping results, with a 100% concordance rate. The probe and primer sequence were listed in **Table S2**.

### Cell Culture and Transfection

Human CRC cells (HT29 and HCT116) were purchased from the American Type Culture Collection (VA, USA) and grown in complete Dulbecco’s modified Eagle medium (HyClone, UT, USA) at 37°C in 5% CO_2_. The culture medium was supplemented with 10% fetal bovine serum (Sigma, MO, USA), penicillin (100 U/mL), and streptomycin (100 μg/mL) (HyClone). All cells were periodically tested for mycoplasma contamination using the MycAway Color One-Step Mycoplasma Detection Kit (Yeasen, Shanghai, China). All cell lines were authenticated by short tandem repeat analysis and used within 6 months.

The miR-297 mimics and miR-297 inhibitors were synthesized by RiboBio (Guangzhou, China). Three small interfering RNAs (siRNAs) against PVT1 were designed and synthesized by GenePharma (Shanghai, China): si-PVT1-1: 5’- GCUGAGAGGGUUGAGAUCUCUGUUU -3’; si-PVT1-2: 5’- CAGCUGGGCUUGAGAUUCCUGGGAA -3’; si-PVT1-3: 5’- UGGACAGUCUGUGGCUGGGUGGGAA -3’; Negative control: 5’- UUCUCCGAACGUGUCACGUTT-3’. The overexpression plasmid for PVT1 was constructed based on pcDNA-3.1. Approximately 2*10^5^ cells were cultured in each well of sex-well plate 24h before the transfection procedure. Cells were separately transfected with siRNA at a final concentration of 1 μM or 0.5 μg plasmid by using Lipofectamine 2000 reagent (Invitrogen, Carlsbad, CA, USA) following the manufacturer’s protocol. The cells were harvested 48 h after transfection.

### RNA Pull-Down Assay

The RNA pull-down assay was performed as described in a previous study (24). Briefly, the 500-bp PVT1 fragment flanking the rs2278176 C or T allele was amplified and cloned into a pcDNA3.1 vector. Biotin-labeled RNA was *in vitro* transcribed with the Biotin RNA Labeling Mix (Roche, Mannheim, Germany) and T7 RNA polymerase (Roche), and treated with RNase-free DNase I (TaKaRa, Japan). Biotin-labeled RNA (2 μg) was purified using the RNeasy Mini Kit (Qiagen, Inc., CA, USA) and incubated with lysates of CRC cells at room temperature (RT) for 1 h. The mixture was then incubated with streptavidin-agarose beads for 1 h at RT. The bound miR-297 was detected by quantitative real-time polymerase chain reaction analysis.

### Dual-Luciferase Reporter Assay

Reporter plasmids were synthesized based on the reporter vector psi-CHECK-2 (Promega, WI, USA). Briefly, 800 ng reporter plasmids along with miR-297 mimics or inhibitors were co-transfected into cells using the Lipofectamine 2000 reagent (Invitrogen). Luciferase activities were measured with Dual-Luciferase Reporter Assay System (Promega) 24 h later.

### Analysis of Cell Apoptosis

In brief, cells were stained with Annexin V-FITC/PI at room temperature in the dark. The rate of apoptosis was analyzed by Flow cytometry (Thermo Fisher Scientific, USA). All assays were independently performed in triplicates.

### Western Blot Assay

The Western blot assay was performed as described in a previous study (25). Briefly, the membrane was incubated at 4°C overnight with GSTA2 (ab232833, Abcam, MA, USA) and β-actin (AT0001, CMCTAG, CA, USA) primary antibodies and then incubated with secondary antibodies for 1 h at RT. Protein bands were visualized by enhanced chemiluminescence (ECL).

### RNA Immunoprecipitation Assay

RNA immunoprecipitation assay was performed using an EZ-Magna RIP RNA-binding protein immunoprecipitation kit (Millipore, MA, USA). Briefly, the cells were lysed into complete RIPA lysis buffer. The whole-cell lysate was then incubated with RIPA buffer containing magnetic beads conjugated with the human anti-Ago2 antibody (1:50 dilution, Millipore) or negative control immunoglobulin G (IgG) for 6 h. Samples were incubated with proteinase K to remove proteins, and then target RNA was extracted for further study.

### Tissue Microarray (TMA) Construction and Immunohistochemistry

The CRC TMAs were constructed by the National Engineering Center for Biochip (Shanghai, China) as reported previously (26). Duplicate 1.0mm diameter cores of tissue from each sample were punched from paraffin tumor blocks and adjacent colorectal tissues. For GSTA2 immunohistochemistry (IHC), the TMA sections were incubated with the anti-GSTA2 antibody (ab232833, Abcam) overnight at 4°C and then stained with 3,3′-diaminobenzidine (Zhongshan Biotech, Beijing, China). Finally, the staining score of GSTA2 was assessed based on the intensity of staining and percentage of positive staining cells as reported elsewhere (27). The ROC curve was used to obtain the optimum cut-off value of GSTA2 IRS. In addition, the AUC at different cut-off values of GSTA2 IRS for survival time from 1 to 9 years was calculated, respectively. In presence of these conditions, samples with IRS 0-4 and IRS 6-12 were defined as low- and high-expression levels for GSTA2, respectively (**Figure S2**).

### Construction of Colorectal Cancer Cells With Indicated Genotypes

Colorectal cancer cells with indicated genotypes were generated by CRISPR/Cas9 strategy. Briefly, the CRISPR/Cas9 expression vectors, which expressed Cas9, sgRNA (GGCACATACAGCCATCATGA) and puromycin resistant gene, was constructed based on the pX330 vector (YSY BIOTECH, CHINA). CRISPR/Cas9 expression plasmid and ssDNA donor (5’- CCGAGGTGCGCGGGTGACCTTGGCATATACAGCCATCATGATGGTACTTTA -3’) were then co-transfected into HT29 and HCT116 cells (both HT29, and HCT116 Cell were rs2278176 CC genotype) using the Lipofectamine 2000 Reagent (Invitrogen). The culture medium was then replaced with 2 mL of complete medium containing 0.6 μg/mL puromycin (VWR International Pty Ltd.) after 24 hours, and the cells were grown for additional 2 weeks. Positive allele-specific colonies were obtained by limiting dilution and were ultimately verified by DNA sequencing (**Figures S3A, B**). For the negative control group, cells were co-transfected with CRISPR/Cas9 expression plasmid and the same wild-type ssDNA donor carrying with C allele (5’- CCGAGGTGCGCGGGTGACCTTGGCACATACAGCCATCATGATGGTACTTTA -3’).

### Mouse Xenograft Assays

The lentiviruses encoding firefly luciferase were constructed and generated as reported previously (28). Briefly, lentiviral particles were mixed gently, and added to the wild type HT29 and HCT116 colorectal cancer cells (MOI=10, both HT29 and HCT116 Cell were rs2278176 CC genotype). The culture medium was replaced with a complete medium containing 7.5 μg/mL blasticidin S after infection with lentivirus for 24 hours. Then, the medium was replaced every 2 days until all the control cells died, and the cells were grown for additional 2 weeks. Surviving cells were then plated at a density of 1 cell per well in a 96-well plate to obtain colonies derived from single cells. After constructing the rs2278176 mutant HT29 and HCT116 colorectal cancer cells (CT and TT genotypes), cells less than fifteen passage after the initial drug selection were implanted in the flank site of 6-week-old female BALB/c nude mice. The tumor growth was assayed 14 days after infection with the IVIS Spectrum Imaging System (Xenogen, USA).

### RNA Extraction and RT–qPCR Analysis

Total RNAs were isolated with the TRIzol reagent (Invitrogen, Carlsbad, CA, USA). Furthermore, 1 μg total RNA was used to synthesis the cDNA according to the manufacturer’s protocol (Takara, Kusatsu, Japan). Glyceraldehyde 3-phosphate dehydrogenase (GAPDH) was used as internal controls in SYBR^®^ Green Realtime PCR Master Mix-Plus kit (Toyobo, Osaka, Japan). Each experiment was performed at least three times in triplicate.

### Statistical Analysis

The endpoints of this study were PFS and OS. PFS was defined as the time from treatment initiation to the first observation of disease progression or death due to any cause. Patients were censored at the time of the last follow-up if no events were observed. OS was defined as the time from the treatment initiation to the date of death due to any cause or censored on the date of the last contact if alive. The log-rank test was used to assess the survival time in different subgroups classified by demographic characteristics, clinical information, and tumor response. The association between the PVT1 polymorphisms and response rate was evaluated using the odds ratios (ORs) and 95% confidence intervals (CIs). The Kaplan–Meier method and log-rank test were used to assess the association between the survival time and the PVT1 polymorphism. Hazard ratios (HRs) and 95% CIs was calculated by univariate or multivariate Cox regression analysis after adjustments for age, gender, location (colon or rectum), grade (low or intermediate/high differentiation), and clinical stage (I - IV). All the tests were two-sided, and a P-value < 0.05 was considered statistically significant. All statistical analyses were carried out using SAS software (version 9.1.3, SAS Institute, NC, USA).

## Results

### Patient Characteristics

**Figure 1:**
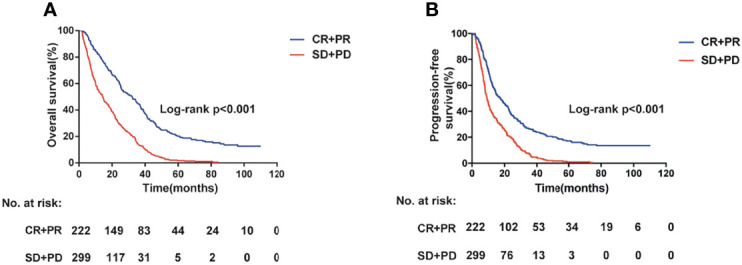
Overall survival **(A)** and progression-free survival **(B)** information for the patients with colorectal cancer having different tumor response status (log-rank *P* < 0.001).

**Table 1** summarizes the demographic and clinical information of the colorectal cancer cases. Briefly, the response rate (CR or PR) was 60.0% and 56.1% in the Xuzhou and Nanjing cohorts, respectively (**Table 1**). The survival information for the patients with CRC is shown in **Table S1**. Maximum follow-up, median PFS, and median OS were 107.5, 11.8, and 19.2 months in the Xuzhou cohort, and 110, 11.7, and 21.9 months in the Nanjing cohort, respectively. Age and tumor response of patients with CRC were associated with OS and PFS (**Figures 1A, B**, and **Table S1**) (both log-rank *P* < 0.05). However, patients with a low grade of differentiation showed increased OS and PFS in the Xuzhou cohort.

### SNP Selection in PVT1

The detailed progress of selecting PVT1 SNPs was exhibited in **Figure S1A**. Briefly, a total of 188 SNPs were firstly screened in PVT1 from the lncRNA SNP database (http://bioinfo.life.hust.edu.cn/lncRNASNP/). We then used the Chinese Han population in Beijing (CHB) population data from the 1000 Genomes Project (March 2012) to screen SNPs minor allele frequency (MAF) > 0.1(n=12 SNPs). After conducting a functional analysis, 2 putative functional SNPs were finally included in our study.

### PVT1 rs2278176 T Allele Increases Survival Time and Predicts Favorable Clinical Outcome in Metastatic Colorectal Cancer Treated With FOLFOX

**Figure 2:**
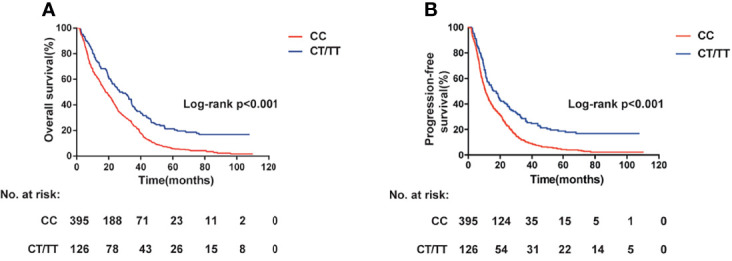
PVT1 rs2278176 genotypes and colorectal cancer: overall survival **(A)** and progression-free survival **(B)** (log-rank *P* < 0.001).

We next assessed the association between the candidate SNPs in lncRNA PVT1 and clinical outcome of patients with CRC. For rs2278176, the call rates were 100% in both Xuzhou and Nanjing Cohort. The genotype frequencies of rs2278176 polymorphism was consistent with Hardy-Weinberg equilibrium (*P* = 0.0583, and *P* = 0.1203 for Xuzhou and Nanjing Cohort, respectively). As shown in **Figures 2A, B** and **Table 2**, the CT/TT genotype was associated with an increased OS (adjusted HR = 0.60, 95% CI = 0.40–0.90 for Xuzhou cohort; adjusted HR = 0.58, 95% CI = 0.44–0.76 for Nanjing cohort; Log-rank *P* < 0.001, adjusted HR = 0.58, 95% CI = 0.47–0.72 for combined cohort) and PFS (adjusted HR = 0.59, 95% CI = 0.40–0.88 for Xuzhou cohort; adjusted HR = 0.58, 95% CI = 0.45–0.76 for Nanjing cohort; Log-rank *P* < 0.001, adjusted HR = 0.58, 95% CI = 0.47-0.73 for combined cohort) compared with the CC genotype.

**Table 2:** Association between PVT1 rs2278176 and colorectal cancer patient’s clinical outcome.

Genotype	Tumor response				OS			PFS		
	CR+PR (%)	CD+PD (%)	P	Adjusted OR (95%CI) ^a^	Median months	log-rank P	Adjusted HR (95% CI) ^a^	Median months	log-rank P	Adjusted HR (95% CI) ^a^
Xuzhou Cohort										
CC	47 (69.1)	83 (81.4)	0.045	1	17.483	0.0005	1	11.267	0.0006	1
CT	16 (23.5)	18 (17.7)		0.60 (0.26-1.37)	19.35		0.71 (0.47-1.08)	11.95		0.69 (0.46-1.04)
TT	5 (7.4)	1 (1.0)		0.13 (0.01-1.30)	76.367		0.13 (0.03-0.56)	61.44		0.19 (0.06-0.64)
CT/TT	21 (30.9)	19 (18.6)	0.065	0.51 (0.23-1.11)	26.083	0.0009	0.60 (0.40-0.90)	16.75	0.001	0.59 (0.40-0.88)
C	110 (80.9)	184 (90.2)	0.0139	1						
T	26 (19.1)	20 (9.8)		0.46 (0.25-0.86)^b^						
Nanjing Cohort										
CC	108 (70.1)	157 (79.7)	0.0235	1	19.6	0.0003	1	10.667	0.0004	1
CT	38 (24.7)	38 (19.3)		0.71 (0.42-1.19)	25.438		0.62 (0.47-0.82)	14.817		0.62 (0.47-0.81)
TT	8 (5.2)	2 (1.0)		0.15 (0.03-0.76)	41.29		0.38 (0.17-0.82)	27.607		0.40 (0.19-0.87)
CT/TT	46 (29.9)	40 (20.3)	0.0387	0.61 (0.37-1.00)	31.012	0.0001	0.58 (0.44-0.76)	16.372	0.0002	0.58 (0.45-0.76)
C	254 (82.5)	352 (89.3)	0.0085	1						
T	54 (17.5)	42 (10.7)		0.56 (0.36-0.87)^b^						
Combined										
CC	155 (69.8)	240 (80.3)	0.0012	1	18.153	<.0001	1	10.909	<.0001	1
CT	54 (24.3)	56 (18.7)		0.66 (0.43-1.02)	23.937		0.63 (0.50-0.80)	14.53		0.63 (0.50-0.80)
TT	13 (5.9)	3 (1.0)		0.15 (0.04-0.54)	49.265		0.31 (0.16-0.59)	38.815		0.33 (0.18-0.64)
CT/TT	67 (30.2)	59 (19.7)	0.0059	0.56 (0.38-0.85)	27.833	<.0001	0.58 (0.47-0.72)	16.698	<.0001	0.58 (0.47-0.73)
C	364 (82.0)	536 (89.6)	0.0004	1						
T	80 (18.0)	62 (10.4)		0.53 (0.37-0.75)^b^						

CR, complete response; PR, partial response; SD, stable disease; PD, progressive disease.

a Adjusted for age, gender, location (Colon or Rectum), grade (Low or Intermediate/High differentiated), depth of invasion, and lymph node metastasis.

b The result did not adjusted for any variable.

Inspired by the results of survival analysis, we then evaluated the association of rs2278176 and tumor response of patients with CRC treated with FOLFOX. Results showed that patients harboring CT/TT genotypes had a greater chance of achieving CR and PR compared with patients harboring the CC genotype in both Xuzhou and Nanjing cohort (adjusted OR = 0.51, 95% CI = 0.23–1.11 for Xuzhou cohort; adjusted OR = 0.61, 95% CI = 0.37–1.00 for Nanjing cohort; adjusted OR = 0.56, 95% CI = 0.38–0.85 for combined cohort). In addition, we also observed a similar result in patients with C allele compared with T allele (adjusted OR = 0.46, 95% CI = 0.25–0.86 for Xuzhou cohort; adjusted OR = 0.56, 95% CI = 0.36–0.87 for Nanjing cohort; adjusted OR = 0.53, 95% CI = 0.37–0.75 for combined cohort) (**Table 2**). However, no significant difference was noted between rs11604 polymorphism and clinical outcome in patients with CRC (**Table S3**).

### PVT1 rs2278176 T Allele Enhances 5-Fu Induced Cell Apoptosis *In Vitro*, and Reduces Tumor Burden *In Vivo* in 5-Fu Treated Nude Mice

To determine the potential molecular mechanisms underlying the results mentioned above, we then constructed HT29 and HCT116 cells with different rs2279276 genotypes (CC, CT, and TT) using the CRISPR-cas9 strategy (**Figures S3A**–**C**). Using the CRISPR-cas9 strategy, we generated 65, 20, and 3 clones for HT29 CC, CT, and TT genotype respectively. In addition, we generated 78, 12, 5 clones for HCT116 CC, CT, and TT genotype, respectively (**Figure S3C**). Finally, three clones from each genotype (CC, CT, and TT) were selected for further functional study.

**Figure 3:**
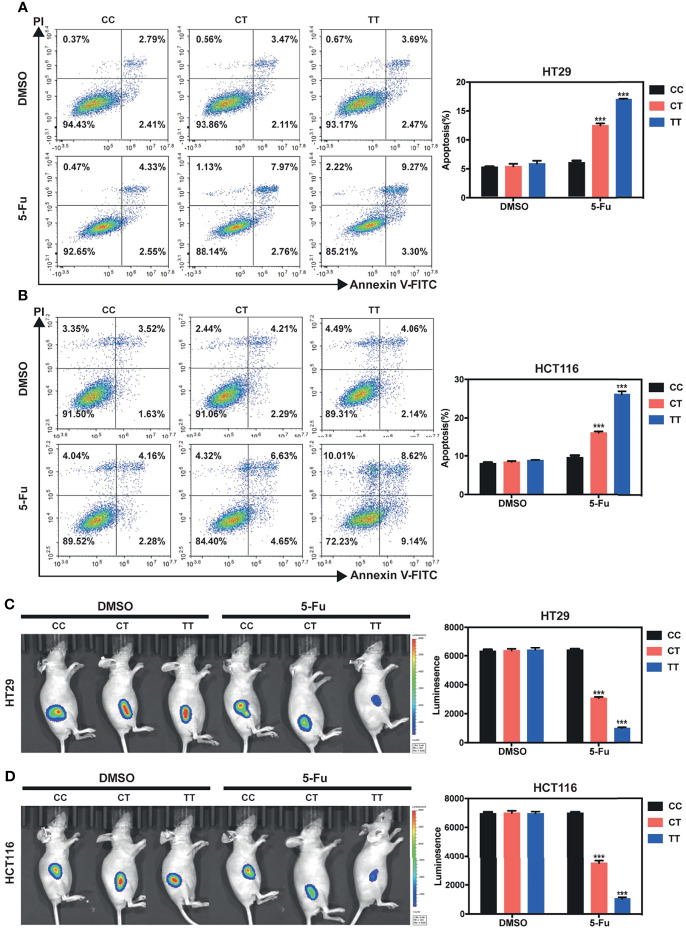
PVT1 rs2278176 T allele enhances 5-Fu induced cell apoptosis *in vitro*, and reduces tumor burden *in vivo* in 5-Fu treated nude mice. **(A, B)** Cell apoptosis in HT29 **(A)** and HCT116 **(B)** cells carrying different rs2278176 genotypes. (*n* = 3 independent clones in each genotype, ***P < 0.001, compared with the CC genotype cells treated with DMSO control, one-way ANOVA; error bars: Standard Error of Mean (SEM)). **(C, D)** Flank tumor burden measured by luciferase activity in nude mice treated with HT29 **(C)** and HCT116 **(D)** cells carrying different rs2278176 genotypes. [*n* = 9 from three independent clones in each genotype, ***P < 0.001, compared with the CC genotype cells treated with DMSO control, one-way ANOVA; error bars: Standard Error of Mean (SEM)].

To seek whether PVT1 rs2278176 C to T mutation would affect CRC cell phenotypes, we initially examined the effects of rs2278176 polymorphism on CRC cells treated with 5-FU (one of the drugs in the FOLFOX regimen). The Flow cytometry and TUNEL assay showed that the apoptotic rate was significantly enhanced by treatment with 5-FU in the CRC cells carrying with the CT/TT genotypes, compared to the DMSO control (**Figures 3A, B** and **Figure S4A**). Furthermore, increased levels of cleaved caspase-3 and cleaved caspase-9 proteins were confirmed in the 5-FU treated CT/TT genotypes CRC cells, relative to the control (**Figure S4B**). *In vivo*, rs2278176 C to T mutation resulted in reduced primary tumor growth in 5-Fu treated colorectal cancer cells (**Figures 3C, D**). Taken together, these results demonstrate that rs2278176 C to T mutation sensitized CRC cells to 5-Fu chemotherapy.

### PVT1 rs2278176 T Allele Alters the Binding Affinity With has-miR-297

**Figure 4:**
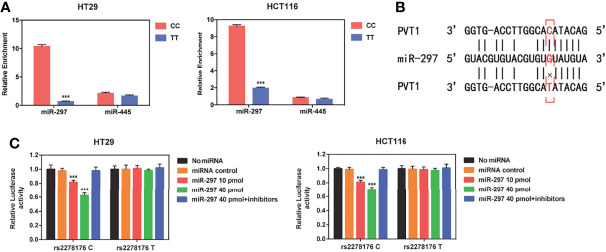
PVT1 rs2278176 T allele alters the binding affinity with has-miR-297. **(A)** Binding affinity of hsa-miR-297 and hsa-miR-455 to PVT1 in wild type (CC) and mutant (TT) HT29 and HCT116 cell lines (*n* = 3 independent clones in each genotype, ***P < 0.001, compared with the CC genotype cells, two-sided *t*-tests; error bars: SD). **(B)** Schematic image of binding interaction between hsa-miR-297 and PVT1 rs2278176 C and T allele. **(C)** Relative reporter gene activity of psi-CHECK-2-PVT1-C-allele or T-allele construct in wild type HT29 and HCT116 cell lines. (*n* = 3 each, ***P < 0.001, compared with the psi-CHECK-2-PVT1-C-allele constructs co-transfected with miRNA control, two-sided *t*-tests; error bars: SD).

Using the bioinformatics algorithm (LncRNASNP database), we found that rs2278176 variant might alter the binding ability for has-miR-297 and has-miR-455-5p to PVT1. To verify the hypothesis, pull-down experiments were performed using biotin-labeled PVT1 probe in CRC cells. Results showed that the binding affinity of has-miR-297 to PVT1 rs2278176 TT genotype was decreased when compared with the PVT1 CC genotype (**Figures 4A, B** **)**. Two luciferase reporter gene plasmids with an rs2278176 C or T allele were further constructed and transfected into the wild type of CRC cells, followed by treatments with has-miR-297 mimics or inhibitors. The data indicated that the luciferase activity of cells with rs2278176 C allele and has-miR-297 mimics reduced compared with negative controls, and this effect was reversed by the has-miR-297 inhibitors (**Figure 4C**). However, no significant change was observed in cells with rs2278176 T allele and has-miR-297 mimics or inhibitors (**Figure 4C**).

### PVT1 Carrying With rs2278176 C Allele Upregulates GSTA2 in an Ago2-Dependent Manner Through Binding hsa-miR-297

**Figure 5:**
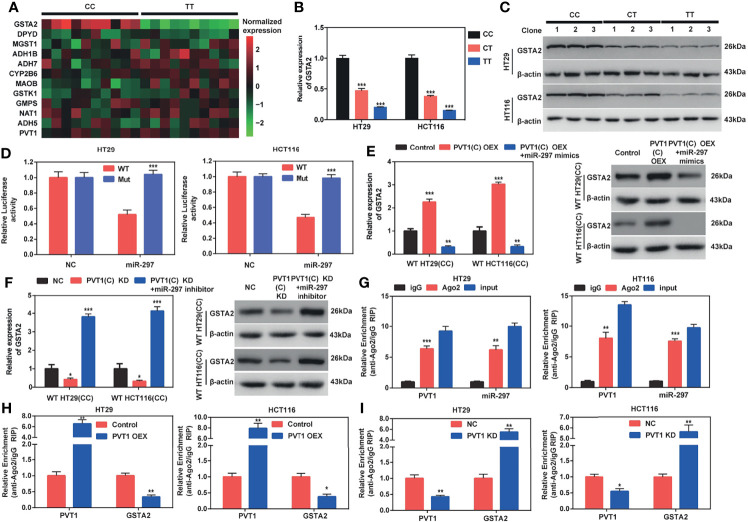
PVT1 carrying with rs2278176 C allele regulates GSTA2 in an Ago2-dependent manner through binding hsa-miR-297. **(A)** Heat map of the expression levels of PVT1 and 11 genes involved in the drug metabolism pathway in tumor tissues of CRC patients harboring CC and TT genotypes. The low and high expression levels are denoted by green and red colors, respectively (*n* = 10 for each genotype). **(B, C)** GSTA2 mRNA and protein levels in HT29 and HCT116 cells carrying different rs2278176 genotypes (*n* = 3 independent clones in each genotype, ^***^ *P* < 0.001, compared with CC genotype, one-way ANOVA; error bars: SEM). **(D)** Relative reporter gene activity of psi-CHECK-2 containing mutant or wild-type GSTA2 3′-UTR co-transfected with miRNA control or hsa-miR-297 mimics in wild type HT29 and HCT116 cell lines (*n* = 3 each, ^***^ *P* < 0.001, compared with the psi-CHECK-2 containing wild-type GSTA2 3′-UTR, two-sided *t*-tests; error bars: SEM). **(E, F)** Relative expression of GSTA2 mRNA and protein in wild type HT29 and HCT116 cell lines co-transfected with PVT1(C allele) overexpression plasmid and hsa-miR-297 mimics **(E)** or PVT1 siRNA and hsa-miR-297 inhibitor **(F)** (*n* = 3 each, ^***^ *P* < 0.001, compared with the cells transfected with empty vector or negative control siRNA, one-way ANOVA; error bars: SEM). **(G)** Relative enrichment of PVT1 and hsa-miR-297 on Ago2 in wild type HT29 and HCT116 cell lines (*n* = 3 each, **P < 0.01, ***P < 0.001, compared with IgG, two-sided *t*-tests; error bars: SEM). **(H, I)** Relative enrichment of PVT1 and GSTA2 on Ago2 in wild type HT29 and HCT116 cell lines transfected with PVT1(C allele) overexpression plasmid **(H)** or PVT1 siRNA **(I)** (*n* = 3 each, ^*^ *P* < 0.05, ^**^ *P* < 0.01, compared with the cells transfected with empty vector or negative control siRNA, two-sided *t-*tests; error bars: SEM).

To further investigate the potential genes that affected the chemosensitivity of patients with CRC harboring different genotypes to FOLFOX, the hsa-miR-297 target genes were predicted using TargetScan. Then, the top 3000 genes were annotated with the Kyoto Encyclopedia of Genes and Genomes pathway database. Interestingly, a total of 11 genes were enriched in the drug metabolism pathway. Then, the expression levels of those 11 genes were detected in cancer (**Figure 5A**) and adjacent normal (**Figure S5A**) tissues collected from CRC patients carrying with CC and TT genotypes (n = 10 for each genotype). The results showed that GSTA2 levels were downregulated in CRC tissues with the TT genotype (*P* < 0.001), but not the levels of the other 10 genes (**Figure 5A**). We did not find any change of these 11 genes in normal tissues from CRC patients with different rs2278176 genotypes (**Figure S5A**). In addition, the PVT1 rs2278176 T allele blunted GSTA2 expression in both mRNA and protein levels in CRC cells (**Figures 5B, C**). We then detected the PVT1 levels in CRC cancer tissues with CC and TT genotypes, and the results showed that the expression levels of PVT1 in patients with different PVT1 rs2278176 genotypes were not significantly altered (**Figures 5A**, **S5A**). These results indicated that the genotype-specific difference in GSTA2 levels is unrelated to PVT1 levels. Thus, it was speculated that GSTA2 might affect the chemosensitivity of patients with CRC to FOLFOX.

A dual-luciferase reporter assay was performed to elucidate whether hsa-miR-297 bound to the 3′- Untranslated Region (3′- UTR) of GSTA2 mRNA (**Figure S5B**). The data showed that hsa-miR-297 mimic dramatically blunted luciferase activities of the CRC cells transfected with the wild-type GSTA2 reporter gene vector, but not the hsa-miR-297-binding sites of the mutant one (two binding motifs for hsa-miR-297 in 3′-UTR of GSTA2 mRNA were mutated) (**Figure 5D**). In addition, the study found that the ectopic expression of wild-type PVT1(C allele) dramatically augmented the expression levels of GSTA2 in wild type HT29 and HCT116 cells (CC genotype), and this elevation could be blunted using hsa-miR-297 mimics (**Figure 5E**). Further, our data showed both mRNA and protein levels of GSTA2 were decreased by co-overexpression of PVT1(T allele) and hsa-miR-297 in mutant type HT29 and HCT116 cells (TT genotype), but not PVT1(T allele) overexpression alone (**Figure S5C**).

To seek the effect of PVT1 knockdown on GSTA2 expression, we then transfected CRC cells with PVT1-siRNAs. The expression levels of PVT1 following siRNA transfection were then confirmed by qPCR and results showed that si-PVT1-1 had the highest knockdown efficiency among three siRNAs (**Figures S5D, E**). Therefore, si-PVT1-1 was selected for further experiments. We found that the inhibition of wild type PVT1(C allele) attenuated the expression level of GSTA2, whereas hsa-miR-297 inhibitor rescued the inhibition in wild-type HT29 and HCT116 cells (CC genotype) (**Figure 5F**). Double knockdown of PVT1(T allele) and hsa-miR-297 increased GSTA2 expression in mutant type HT29 and HCT116 cells (TT genotype), but not PVT1(T allele) blunt alone (**Figure S5F**). Together, these results corroborated that PVT1 carrying with rs2278176 C allele regulated GSTA2 through binding with hsa-miR-297.

MicroRNAs repressed translation and degraded target mRNA in an Ago2-dependent manner by binding to their targets (22). To determine whether PVT1 could bind to hsa-miR-297 in this way, a RIP assay was performed on Ago2 in wild-type HT29 and HCT116 cells (CC genotype). The results showed that PVT1 and hsa-miR-297 were substantially enriched with the anti-Ago antibody compared with the anti-normal IgG antibody (**Figure 5G**). Moreover, the overexpression of PVT1(C allele) led to an increased enrichment of Ago2 on PVT1 but decreased enrichment on GSTA2 (**Figure 5H**). In parallel, the knockdown of PVT1 led to opposite effects (**Figure 5I**). Taken together, these results demonstrated that PVT1 carrying with rs2278176 C allele regulates GSTA2 in an Ago2-dependent manner through binding hsa-miR-297.

### High GSTA2 Expression Predicts Poor Clinical Outcome in Metastatic Colorectal Cancer Treated With FOLFOX

**Figure 6:**
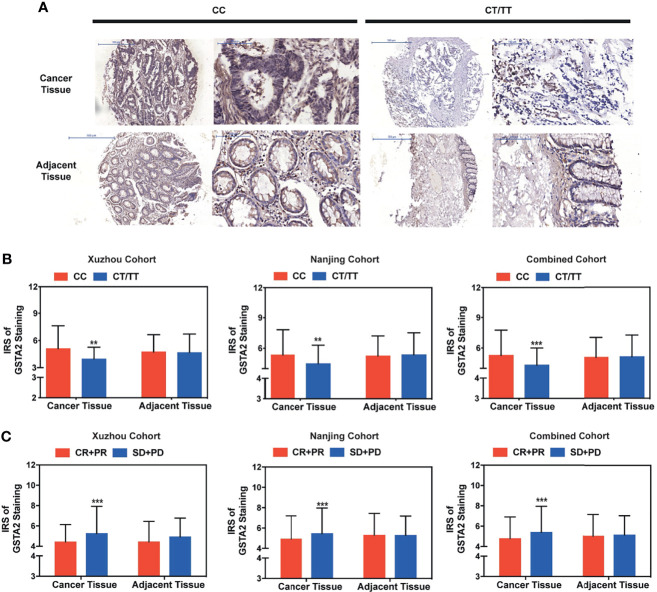
High GSTA2 expression predicted poor clinical outcome in metastatic colorectal cancer treated with FOLFOX. **(A)** Immunohistochemical staining for GSTA2 in patients with colorectal cancer patients harboring different rs2278176 genotypes (representative IHC images were obtained at 100× and 400× magnification). **(B)** The IRS of the expression of GSTA2 in patients with colorectal cancer harboring different rs2278176 genotypes (*n* = 149, 312, and 461 in Xuzhou, Nanjing, and combined cohort, respectively, ^***^ *P* < 0.001; two-sided *t*-tests; error bars: SD). **(C)** The IRS of GSTA2 expression in colorectal patients with different tumor response status. (*n* = 149, 312, and 461 in Xuzhou, Nanjing, and combined cohort, respectively, ^***^ *P* < 0.001; two-sided *t*-tests; error bars: SD). **P < 0.01.

To further explore whether the rs2278176 polymorphism regulated the expression of GSTA2, the tissue microarrays (TMAs) were set up with 5-year survival and tumor response information from colorectal cancer and adjacent tissues in the Xuzhou and Nanjing cohorts. The expression levels of GSTA2 were evaluated in patients with CRC harboring different PVT1 rs2278176 genotypes using immunohistochemistry. Finally, a total of 461 CRC patients were enrolled for final analysis as some of them lost in antigen retrieval process (n=130, 34, 6 in CC, CT, TT genotype for Xuzhou cohort, respectively, and n=265,76, 10 in CC, CT, TT genotype for Nanjing cohort, respectively). The results showed that GSTA2 levels were downregulated in the cancer tissues of patients with the PVT1 rs2278176 CT/TT genotype, but not adjacent tissues (**Figures 6A, B** **)**. In addition, higher expression levels of GSTA2 were observed in the cancer tissues of patients with SD and PD, but not adjacent tissues (**Figure 6C** and **Table S4**). Further survival analysis showed that high GSTA2 levels were also associated with decreased OS and PFS (**Tables S4**).

## Discussion

This study explored the relationship between two SNPs in the lncRNA PVT1 and clinical outcome of patients with metastatic CRC treated with FOLFOX. The results demonstrated that the rs2278176 CT/TT genotype was associated with greater efficacy of FOLFOX chemotherapy response and increased survival times in patients with metastatic CRC. Mechanistically, PVT1 rs2278176 C to T mutation might alter the binding affinity of hsa-miR-297 to PVT1, leading to the low expression level of GSTA2, ultimately enhancing the chemosensitivity of patients with CRC to FOLFOX.

FOLFOX (oxaliplatin, 5-fluorouracil, and leucovorin) regimen is one of the standards first-line chemotherapies for metastatic CRC (29, 30). However, in clinical practice, only a minority of patients benefited from this treatment, suggesting individual differences in response to FOLFOX treatment. Accumulating evidence suggested that SNPs might affect responses to anticancer drug treatment, including CRC (19–21). Chen et al. showed that the rs3742106 TT genotype in the 3′-UTR of ABCC4 gene might promote the binding with miR-3190-5p, in turn inhibiting the expression of ABCC4 protein, thus leading to improved sensitivity of CRC cells to chemotherapy with 5-FU (19). Another previous study showed that SNPs in the insulin-like growth factor axis were associated with tumor response in patients with CRC treated with FOLFOX chemotherapy (31). The present study found that patients with CRC carrying the PVT1 rs2278176 CT/TT genotype have greater clinical efficacy for FOLFOX-based chemotherapy. These findings further corroborated the evidence that SNPs could be used to predict the response to FOLFOX-based chemotherapy.

The human PVT1 gene is located on chromosome 8q24 and encodes a lncRNA, which is involved in a variety of cancer types and plays a major role in cancer development and metastasis (10–13). Tseng et al. reported that PVT1 could protect the MYC protein from degradation by decreasing phosphorylation of its threonine residue 58, thereby promoting cancer cell proliferation (32). Takahashi et al. showed that PVT1 could inhibit the apoptosis of CRC cells *via* TGF-β signaling activation, ultimately contributing to the risk of CRC (33). Recently, SNPs located on lncRNAs have been reported to be functionally important in creating or eliminating a miRNA-binding site (34–36). In this study, we found that the rs2278176 variant might alter the binding ability for hsa-miR-297 and hsa-miR-455-5p on PVT1 based on the LncRNASNP database. Moreover, it has been shown that PVT1 regulated CRC development by acting as a competing endogenous RNA (ceRNA) for miR-455-5p (37). However, only hsa-miR-297 was confirmed to bind with PVT1 using pull-down experiments in our study, but not hsa-miR-455-5p. This result might be due to the weak binding affinity between PVT1 and miR-455-5p. It has been reported that miR-297 was downregulated in HCT-116/L-OHP cells. However, overexpression miR-297 in multidrug resistance CRC cells sensitized these cells to anti-cancer drugs *in vitro* and *in vivo* through regulated MRP-2 expression (38). The present study found that the rs2278176 variant might alter the binding site for hsa-miR-297, thereby influencing the expression levels of GSTA2. These results further indicated that miR-297 might play an important role in the anticancer drug treatment of CRC.

Glutathione S-transferases are a family of phase II detoxification enzymes involved in the detoxification of electrophilic compounds by conjugation with glutathione (39). In addition, glutathione conjugation leads to the greater water solubility of these compounds, promoting the elimination of toxic compounds (39, 40), ultimately contributing to drug resistance in cancer therapies (40). The present study demonstrated that PVT1 might act as a ceRNA and a natural sponge for hsa-miR-297 to regulate the expression of GSTA2. Moreover, this effect might be influenced by rs2278176 polymorphism. Taken together, these findings further corroborated that the rs2278176 polymorphism could be used to predict the response to FOLFOX-based chemotherapy.

Undeniably, some limitations should be addressed in our study. First, the sample size of our study might not be large enough to achieve sufficient statistical power, especially for the stratified analyses. Second, all the enrolled subjects belong to the Chinese population. Thus, further studies with enough samples and different ethnic populations are required to confirm our findings. Third, we assessed the levels of a gene involved in the drug metabolism pathway only by using qRT-PCR. Other detection methods, such as western blot, are needed to confirm these results.

## Data Availability Statement

The original contributions presented in the study are included in the article/**Supplementary Material**. Further inquiries can be directed to the corresponding authors.

## Ethics Statement

The studies involving human participants were reviewed and approved by Institutional Review Board of Southeast University. The patients/participants provided their written informed consent to participate in this study. The animal study was reviewed and approved by The Institutional Animal Care and Use Committee of Southeast University.

## Author Contributions

SW and RC conceived of the study. SW and XY performed the experiments. WT collected clinical samples. SW, XL, and RC analyzed the data and wrote the paper. GF, MR, MA, and RC reviewed and revised the paper. All authors read and approved the final manuscript.

## Funding

This work was financially supported by Fund of International Cooperation and Exchange of the National Natural Science Foundation of China (81861138017), State Key Program of the National Natural Science Foundation of China (81730088), the National Natural Science Foundation of China (82003499), Scientific Research Common Program of Beijing Municipal Commission of Education (KM202110025206), Guangdong Provincial Natural Science Foundation Team Project (2018B030312005), the Ministry of Foreign Affairs and International Cooperation of Italy (PGR00962), the National Institute of Environmental Health Sciences (NIEHS), R01 ES10563, R01 ES07331 and R01 ES020852.

## Conflict of Interest

The authors declare that the research was conducted in the absence of any commercial or financial relationships that could be construed as a potential conflict of interest.

## Publisher’s Note

All claims expressed in this article are solely those of the authors and do not necessarily represent those of their affiliated organizations, or those of the publisher, the editors and the reviewers. Any product that may be evaluated in this article, or claim that may be made by its manufacturer, is not guaranteed or endorsed by the publisher.
