# Supplementary material for: Chemotherapeutic Risk lncRNA-PVT1 SNP Sensitizes Metastatic Colorectal Cancer to FOLFOX Regimen
Source: Front Oncol. 2022 Mar 31;12:808889. doi: 10.3389/fonc.2022.808889 (PMC9008320; doi:10.3389/fonc.2022.808889)
Supplement: Supplementary file 1 [file DataSheet_1.pdf]

Supplementary Material

Supplementary Figures

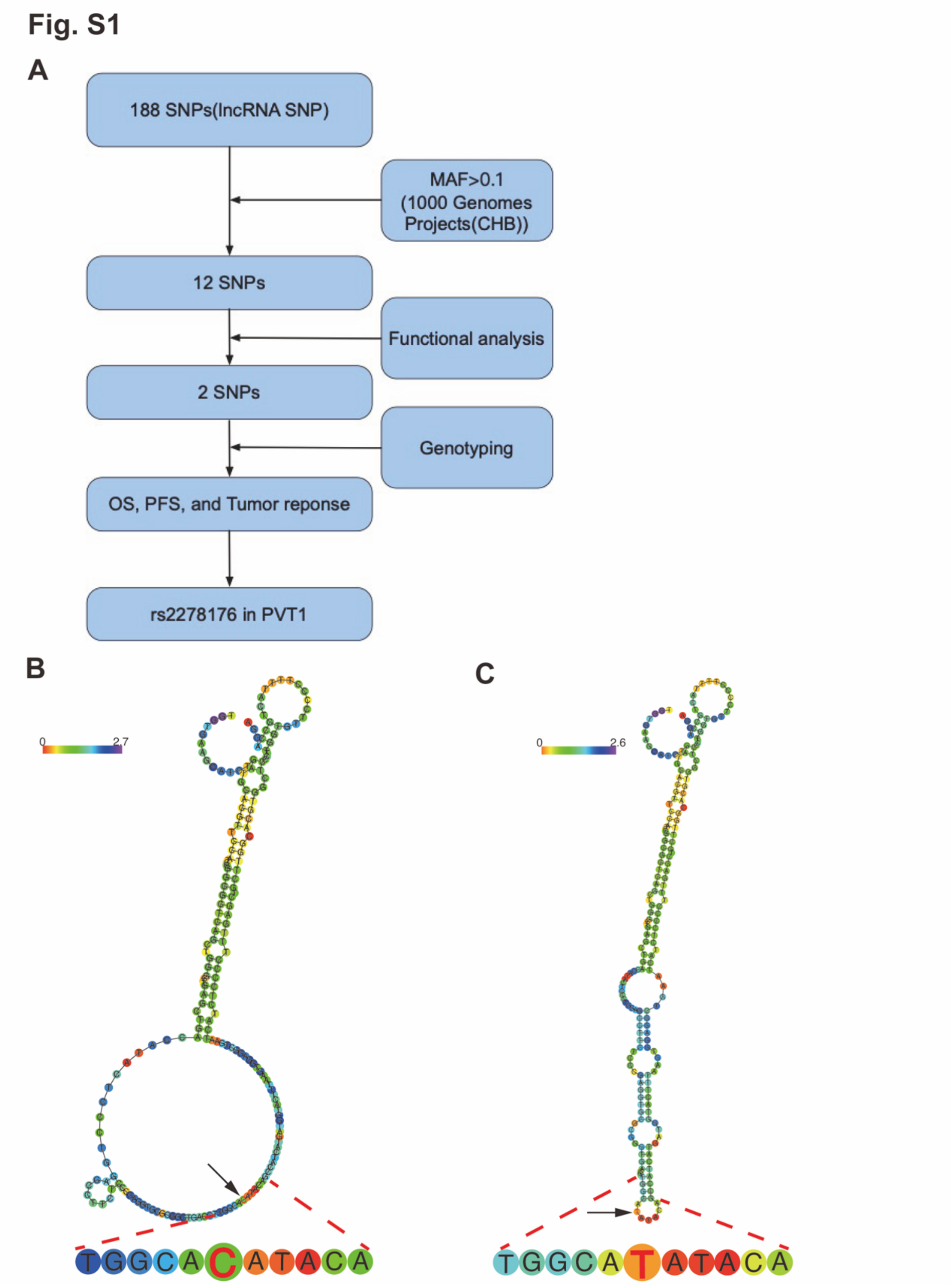

Supplementary Figure 1. SNPs selecting and functional prediction for PVT1 SNPs. (A) Flowchart for selecting SNPs in PVT1. (MAF minor allele frequency; PFS progression free survival; OS overall survival; (B and C) Predicted folding structures for PVT1 with rs2278176 C allele(B) or rs2278176 T allele(C) (as shown by red arrow).

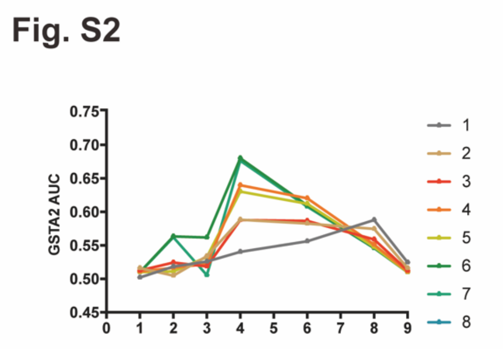

Supplementary Figure 2. Area under the curve at different cutoff values of GSTA2. Immunoreactivity score for overall survival time of 1–8 years.

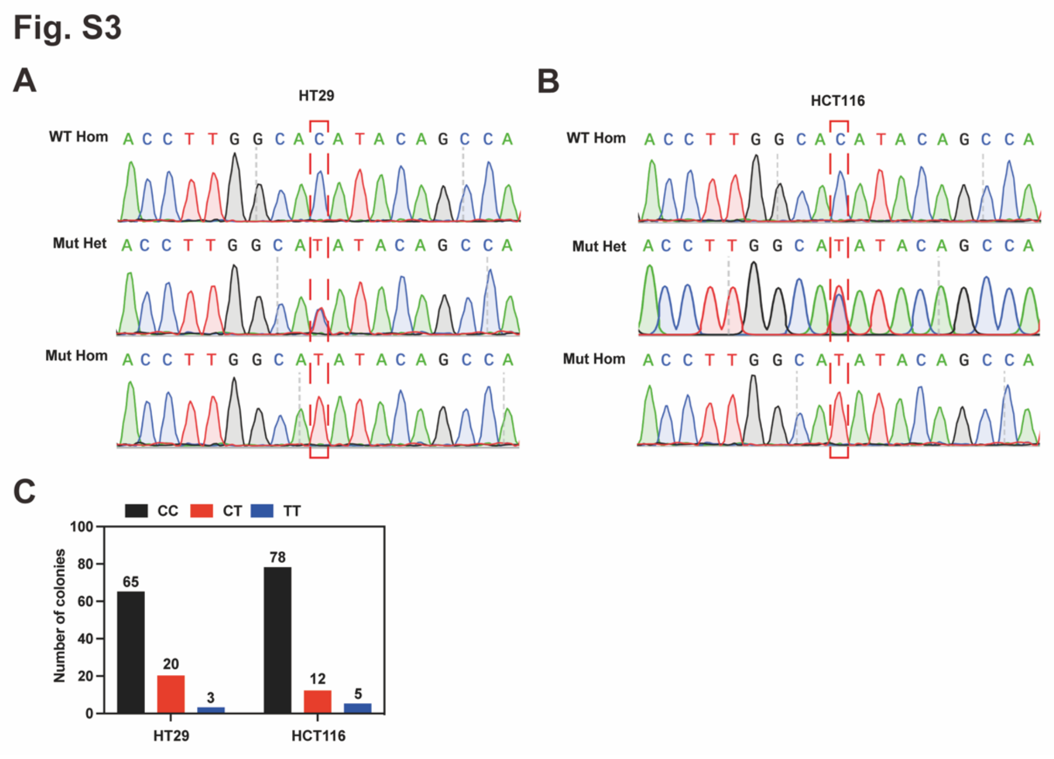

Supplementary Figure 3. Construction of colorectal cancer cells with indicated genotypes. (A-B) Sanger sequencing for wild-type (WT) and mutated (Mut) HT29(A) and HCT116(B) cells. (C)Number of clones generated from CRISPR-cas9 strategy.

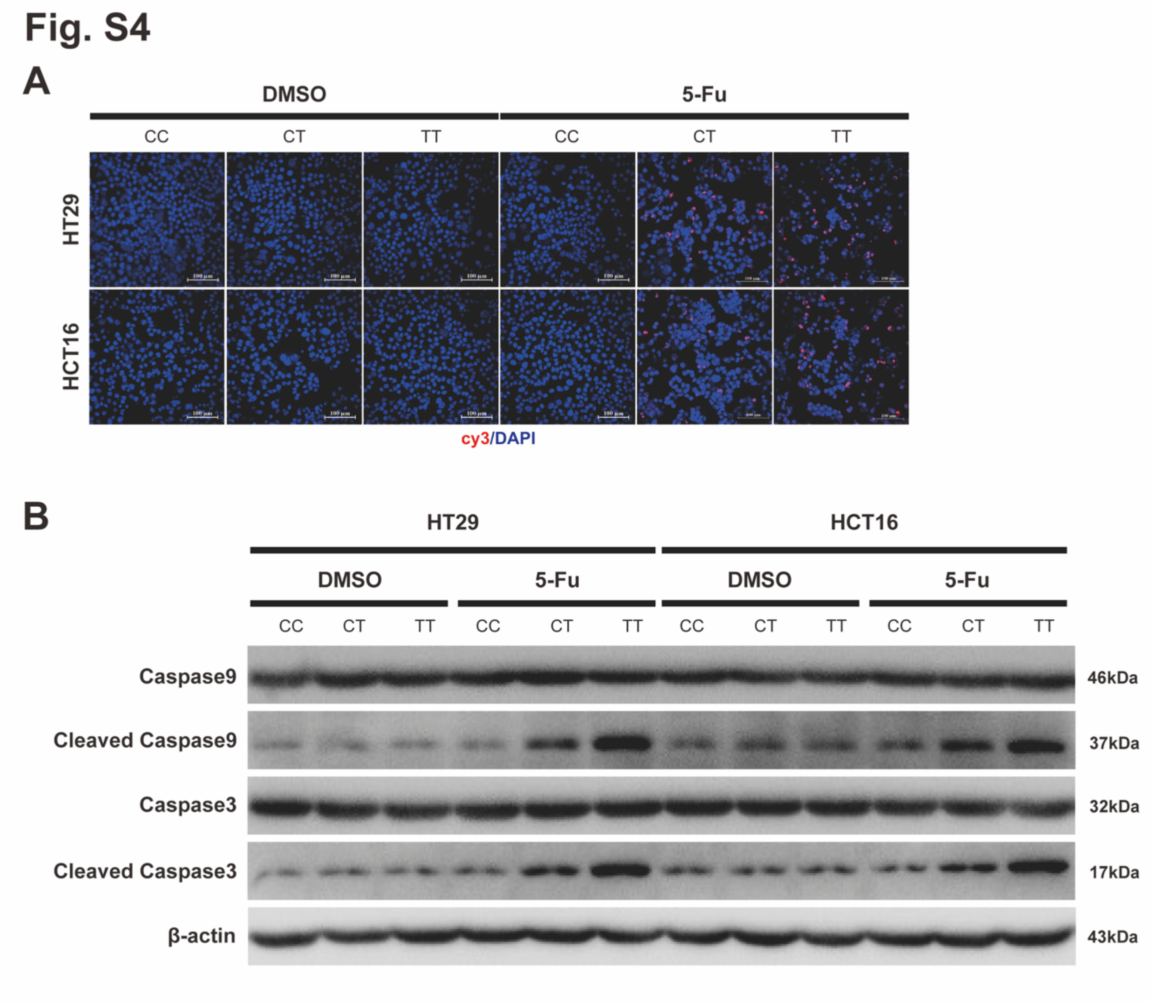

Supplementary Figure 4. Effects of rs2278176 polymorphism on colorectal cancer cell apoptosis. (A) TUNEL staining in DMSO or 5-Fu treated different rs2278176 genotype CRC cells;(B) Effect of DMSO or 5-Fu on protein levels of caspase3, caspase9, cleaved caspase3 and cleaved caspase9 in CRC cells carrying different rs2278176 genotype.

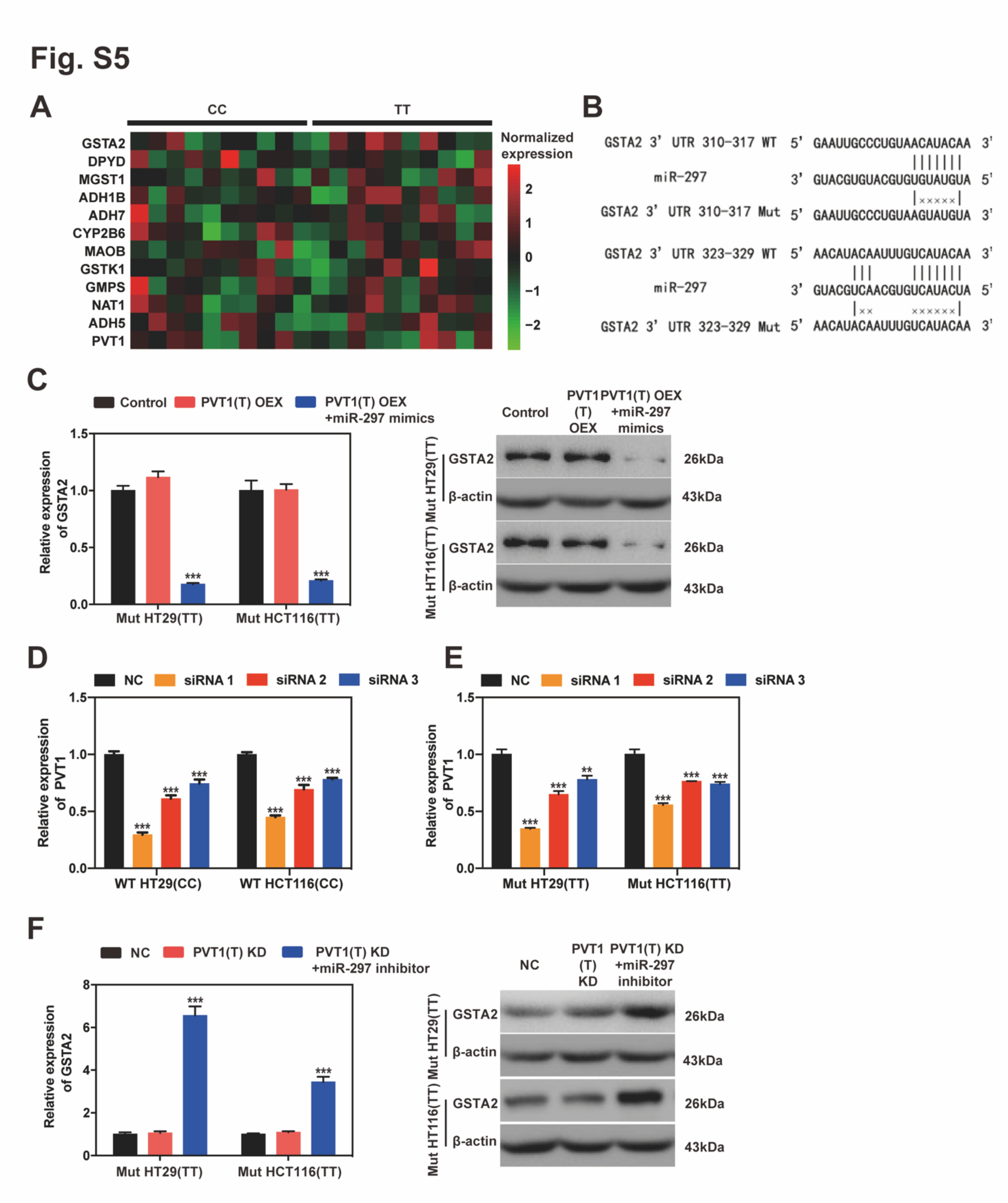

Supplementary Figure 5. GSTA2 was regulated by hsa-miR-297, but not PVT1 carrying with rs2278176 T allele. (A)Heat map of the expression levels of PVT1 and 11 genes involved in the drug metabolism pathway in normal tissues of CRC patients harboring CC and TT genotypes. The low and high expression levels are denoted by green and red colors, respectively (n = 10 for each genotype). (B) Two predicted target sites of hsa-miR-297 on GSTA2 and the mutant sequence are shown. (C)

Relative expression of GSTA2 mRNA and protein in mutant (TT) HT29 and HCT116 cell lines co-transfected with PVT1(T allele) overexpression plasmid and hsa-miR-297 mimics (n = 3 each, ***P < 0.001, compared with the cells transfected with empty vector, one-way ANOVA, error bars, SEM). (D-E) PVT1 expression in WT(D) and Mut(E) HT29 and HCT116 cells treated with siRNAs targeting PVT1(n = 3 each, **P < 0.01, ***P < 0.001, compared with the cells transfected with negative control siRNA, one-way ANOVA, error bars, SEM). (F)Relative expression of GSTA2 mRNA and protein in mutant (TT) HT29 and HCT116 cell lines co-transfected with PVT1 siRNA (T allele) and hsa-miR-297 inhibitor (n = 3 each, ***P < 0.001, compared with the cells transfected with negative control siRNA, one-way ANOVA, error bars, SEM)

Supplementary Tables

Supplementary TableS1 Demographic and survival information for the colorectal cancer patients

Variables	Xuzhou Cohort（N=170）				Nanjing Cohort(N=351)			
	MST (months)	Log-Rank P	MPFST (months)	Log-Rank P	MST (months)	Log-Rank P	MPFST (months)	Log-Rank P
Age		0.0031		0.0079		0.0207		0.0119
≤54	23.300		12.867		21.967		11.133	
＞54	16.700		10.507		21.883		12.096	
Sex		0.3677		0.496		0.664		0.4412
Male	16.35		11.017		21.967		11.958	
Female	19.90		12.586		21.172		11.300	
Location		0.2847		0.363		0.3586		0.3737
colon	19.000		11.267		19.533		10.822	
rectal	19.150		12.586		23.667		12.809	
Grade		<.0001		<.0001		0.6261		0.8225
low	31.083		20.692		23.713		13.371	
intermediate/High	10.200		8.300		19.433		10.667	
Depth of invasion		0.1144		0.1229		0.0384		0.1335
T1	4.533		4.533		12.467		8.030	
T2	20.633		10.167		23.667		13.666	
T3	12.500		10.000		33.687		20.175	
T4	19.300		12.067		19.933		10.767	
Lymph node metastasis		0.1204		0.1723		0.366		0.271
N0	11.233		11.000		23.330		14.533	
N1	19.300		12.067		21.598		11.590	
Tumor response		<.0001		<.0001		<.0001		<.0001
CR+PR	28.883		16.750		32.617		17.520	
SD+PD	11.933		9.467		15.033		9.2220	

Abbreviations: CR, complete response; PR, partial response; SD, stable disease; PD, progressive disease; MST, median of overall survival time; MPFST, median of progression-free survival time.

Supplementary TableS2 Primer and probe information for the rs2278176 andrs11640

SNPs	Primer	Probe
rs2278176	F: TGCTTCTGCAGCTGACCATACT	T: FAM-ACCTTGGCATATACA-MGB
	R: GAGATGATTCAGCCTCCACTTAAAG	C: VIC-TTGGCACATACAGCCA-MGB
rs11604	F: TCACAAGCCCCACCAAGAG	T: FAM-CAGGAATGCTTGGAGG-MGB
	R: CAAGATGCAGTAGCCTCAGTGAA	C: VIC-CCCAGGAACGCTT-MGB

Supplementary TableS3 Association between PVT1 rs11604 and colorectal cancer patient's clinical outcome.

Genotype	Tumor response				OS			PFS		
	CR+PR (%)	CD+PD (%)	P	Adjusted OR (95%CI) [a]	Median months	log-rank P	Adjusted HR (95% CI) [a]	Median months	log-rank P	Adjusted HR (95% CI) [a]
Xuzhou Cohort										
CC	52(76.5)	75(73.5)	0.4938	1	19.3	0.1394	1	11.833	0.108	1
CT	12(19.1)	25(24.5)		1.39(0.62-3.12)	18.4		1.26(0.87-1.84)	10.805		1.23(0.85-1.78)
TT	3(4.4)	2(2.0)		0.45(0.07-2.98)	55		0.54(0.19-1.51)	43.555		0.52(0.20-1.36)
CT/TT	16(23.5)	27(26.5)	0.6656	1.20(0.56-2.57)	19	0.827	1.12(0.78-1.61)	11.233	0.7474	1.08(0.76-1.53)
Nanjing Cohort										
CC	119(77.3)	145(74.6)	0.7045	1	21.483	0.7993	1	10.996	0.9253	1
CT	31(20.1)	45(22.8)		1.21(0.72-2.04)	21.988		1.00(0.76-1.30)	17.754		0.98(0.76-1.27)
TT	4(2.6)	7(2.6)		1.41(0.39-5.03)	34.867		1.27(0.69-2.35)	21.634		1.18(0.64-21.9)
CT/TT	35(22.7)	52(26.4)	0.4296	1.23(0.75-2.03)	22.01	0.7749	1.03(0.80-1.32)	11.775	0.9761	1.00(0.78-1.27)
Combined										
CC	171(77.0)	220(73.6)	0.6183	1	20.177	0.7005	1	11.615	0.5134	1
CT	44(19.8)	70(23.4)		1.25(0.81-1.92)	21.45		1.05(0.85-1.30)	12.346		1.04(0.84-1.28)
TT	7(3.2)	9(3.0)		1.02(0.37-2.85)	36.167		1.01(0.60-1.71)	21.939		0.88(0.53-1.47)
CT/TT	51(23.0)	79(26.4)	0.3684	1.22(0.81-1.83)	21.883	0.6725	1.05(0.85-1.28)	12.754	0.8083	1.02(0.83-1.24)

Abbreviations: CR, complete response; PR, partial response; SD, stable disease; PD, progressive disease.

[a] Adjusted for age, gender, location (Colon or Rectum), grade (Low or Intermediate/High differentiated), depth of invasion, and lymph node metastasis

Supplementary TableS4 Association between GSTA2 expression and response rates, colorectal cancer patient's survival.

Genotype	Tumor response				OS			PFS		
	CR+PR (%)	SD+PD (%)	P	Adjusted OR (95%CI) [a]	Median month	log-rank P	Adjusted HR (95% CI) [a]	Median month	log-rank P	Adjusted HR (95% CI) [a]
Xuzhou Cohort										
low	52（76.5）	64（62.8）	0.0597	1	20.317	0.0037	1	12.586	0.0042	
high	16（23.5）	38（37.3）		1.78(0.85-3.72)	16.350		1.42(1.01-2.01)	9.252		1.46(1.03-2.06)
Nanjing Cohort										
low	99（64.3）	106（53.8）	0.0481	1	26.967	<.0001	1	16.933	<.0001	
high	55（35.7）	91（46.2）		1.53(0.99-2.37)	15.233		1.84(1.47-2.31)	8.724		1.80(1.44-2.26)
Combined										
low	151（68.0）	170（56.9）	0.0096	1	25.067	<.0001	1	14.844	<.0001	
high	71（32.0）	129（43.1）		1.61(1.12-2.32）	15.517		1.68(1.40-2.03)	8.833		1.68(1.39-2.02)

Abbreviations: CR, complete response; PR, partial response; SD, stable disease; PD, progressive disease.

[a] Adjusted for age, gender, location (Colon or Rectum), grade (Low or Intermediate/High differentiated), depth of invasion, and lymph node metastasis.
